# 

**Published:** 1905-07

**Authors:** 


					﻿SUBSCRIPTION $1.00
ATLANTA PUBLISHED MONTHLY.
JOURNAL-RECORD
MEDICINE.
Successor to Atlanta Hedical and Surgical Journal,i£st^)li»hed4tf$j/\^
and Southern] fledical Record, Established 1870.	A
Vol. VII.	Atlanta, Ga., July, 190&	No/ 4.
				

